# Delay Discounting and the Income-Food Insecurity-Obesity Paradox in Mothers

**DOI:** 10.1155/2023/8898498

**Published:** 2023-09-19

**Authors:** Leonard H. Epstein, Ashfique Rizwan, Rocco A. Paluch, Jennifer L. Temple

**Affiliations:** ^1^Department of Pediatrics, Jacobs School of Medicine and Biomedical Sciences, University at Buffalo, Buffalo, NY, USA; ^2^Department of Exercise and Nutrition Sciences, School of Public Health and Health Professions, University at Buffalo, Buffalo, NY, USA; ^3^Community Health and Health Behavior, School of Public Health and Health Professions, University at Buffalo, Buffalo, NY, USA

## Abstract

Food insecurity, defined as unpredictable access to food that may not meet a person's nutritional needs, is paradoxically associated with higher BMI (kg/m^2^) and obesity. Research has shown delay discounting, a behavioral economic measure of the preference for immediate rather than delayed rewards, is related to higher BMI, and moderates the relationship between income and food insecurity. Based on this research, we used regression models to test whether delay discounting, consideration of future consequences, and perceived stress were atemporal mediators of the food insecurity-BMI relation in 313 mothers, controlling for demographic variables. A secondary aim was to replicate the finding that delay discounting moderates the relationship between low income and high food insecurity. Results showed that low income was associated with higher food insecurity, and higher food insecurity was associated with higher BMI. Delay discounting was the only variable that was indirectly related to both paths of the food-insecurity-BMI relation. Delay discounting accounted for 22.2% of the variance in the low-income-food insecurity-obesity relation, and the total model accounted for 38.0% of the variance. The relation between low income and food insecurity was moderated by delay discounting. These data suggest that delay discounting is a potential mediator of the relationship between food insecurity and high BMI, which suggests reducing discounting in the future could be a novel target to reduce food insecurity and help people with food insecurity to reduce their excess body weight. Trial Registration. This trial is registered with NCT02873715.

## 1. Introduction

Food insecurity due to lack of regular access to food that meets their nutritional needs [1, 2] affects many low-income families [3]. Data from the Panel Study of Income Dynamics suggest that the percentage of families with children who experience food insecurity has increased from 8.8% in 1990–2003 to 10.9% in 2015–2019. Data from this study suggest that the experience of food insecurity may change over time, as only 33.1% of low-income families report high food security at both time points, suggesting there are protective factors that reduce the likelihood that a low-income family consistently experiences food insecurity [3].

There is a strong relation between food insecurity and elevated body mass index (BMI = kg/m^2^) and obesity (BMI ≥30), which has led to the food insecurity-obesity paradox [4, 5]. Given that people with food insecurity experience unpredictable access to food, may lack access to healthy food in their environment, and may miss meals and go hungry [4, 6, 7], it is not expected that they would have excess body weight and obesity. This unexpected relation is the basis for the food-insecurity obesity paradox. The relation between food insecurity and excess body weight is stronger for women than men [4, 8]. Many children in low-income families also experience food insecurity [3], and the study of mothers may be particularly interesting as they may be more sensitive to the effects of food insecurity on body weight [4, 8], and they may model behaviors related to weight gain that can influence eating behaviors of their children [9].

Shah and colleagues [10] theorize that resource scarcity that is characteristic of food insecurity creates a mindset in which individuals allocate attention to behaviors that reinforce poverty (e.g., borrowing money) while neglecting others (e.g., failing to enroll in assistance programs). Economic scarcity can lead to a person focusing on immediate needs, including putting food on the table that night or for the week, rather than considering longer-term goals such as saving to pay off debts, purchasing a home, or saving for children's college [11–13]. Low income is related to higher discounting of the future [14, 15], and a narrow temporal window focuses on immediate demands and immediate gratification as an adaptive response to pressing needs and tending to trade-offs that must be considered in meeting those needs [16, 17]. Delay discounting (DD), which measures a person's temporal orientation towards either small, but immediate rewards, or large, delayed rewards [18, 19], is associated with income [14, 15], obesity [20–22], and food insecurity [11–13, 23]. Given that DD is associated with low income [14, 15], high food insecurity [11–13, 23], obesity [20–22], and increases during scarcity conditions [11–15, 23, 24], DD may provide insight into the relation between low income and food insecurity as well as the food insecurity-obesity paradox [4, 5]. This suggests that having a wide temporal window and not discounting the future may be a protective factor for people with low income not experiencing food insecurity [11].

While DD assesses a person's style of decision-making that tends to focus on smaller, immediate rewards versus larger, later rewards, there are other approaches to studying how people make decisions that focus on the balance between immediate and long-term needs. This is the basis for prospective thinking or a person's temporal orientation towards the future rather than the present. A widely used measure of temporal orientation is the Consideration of Future Consequences Scale (CFCS) [25–27], which assesses how much people consider future consequences of their behavior when making a decision about present behavior. It is relevant to consider whether another measure of a person's temporal orientation could help explain the relationship between food insecurity and BMI.

People with low income and food insecurity often experience stress [5, 23, 28]. Stress is associated with obesity [23, 29], and it can increase food reward [30] and can lead to emotional eating to regulate negative affect [31], both of which can lead to positive energy balance, weight gain, and obesity. Stress also impacts cognitive function, including reducing working memory capacity [32], increasing attentional bias for food [33], and increasing DD [23], which can shift decision-making towards immediate gratification and characteristics of people who experience food insecurity [11–13, 23].

The primary goal of this study is to assess whether DD, CFCS, and stress can help understand the relationship between food insecurity and higher BMI in mothers. A secondary goal is replicating the moderating effect of DD on the low-income-high food insecurity relationship that we observed in a previous study in which DD and financial planning moderated the relationship between low income and food insecurity [11]. In that study, people with low income who engaged in some financial planning and did not discount the future showed levels of food insecurity equivalent to people with higher income levels. People with low income who engaged in financial planning with a narrow temporal window, as defined by high levels of DD, experienced high levels of food insecurity, equivalent to not engaging in financial planning. These data suggest that not discounting the future for people with low income was an important style of decision-making that is associated with lower food insecurity.

## 2. Methods

### 2.1. Subjects and Design

This study was approved by the University at Buffalo Institutional Review Board and was conducted online in November 2018. The study was a secondary analysis of data collected for a cross-sectional study of parents recruited from ResearchMatch.org, a crowdsourcing platform. The parent study was designed to study the relationship between stress and parenting. No publications were generated from the analysis of these relations. A recruitment message was sent to randomly selected individuals who met our study target (e.g., adults 18–55). E-mail addresses of those interested in participating were exported from ResearchMatch.org, and potential participants were sent an e-mail with the link to a Qualtrics questionnaire. An IRB-approved consent was presented to the participant upon opening the link, and consent was obtained by the participant indicating (e.g., clicking) “I agree to the terms described above.” 569 people consented and were eligible to participate (e.g., an adult 18–55 with at least one child between 2 and 15), and of those, 369 participants provided full, complete, and valid responses to all of the study questionnaires, including the DD task [34], and 313 of those were mothers, the focus of this analysis.

### 2.2. Measures

Participants completed the MacArthur network socio-demographic questionnaire (e.g., age, gender, marital status, employment status, education, income, race, and perceived social status) [35] as well as the short form, 6-item food insecurity questionnaire [36] that asks questions regarding experience of food insecurity in the last 12 months. Cutoffs for low food security and very low food security are 2 and 5, respectively. Parents self-reported their height and weight, which was used to calculate body mass index (BMI = kg/m^2^). Overweight and obesity are defined as BMI ≥25 and BMI >30, respectively [35]. Previous research has validated the use of height and weight self-reports for observational studies [37]. Income was converted to percent over poverty based on household size using 2018 poverty data [38].

Participants completed an adjusting amount DD task [39]. During this task, participants chose between receiving a larger amount of money at a future time point (1 day, 7 days, 30 days, 182 days, and 365 days) and a smaller amount of money now (e.g., *“Would you rather have $50 now or $100 in one week?*). The amount of money offered “now” adjusted based on participant's prior response. Delayed time choices were presented in a random order. Depending on the participant's response, the immediately available amount was adjusted up (if delayed choice selected) or down (if immediate choice selected). Each time delay included five trials with the amount adjusting half that of the previous adjustment [39]. Patterns of responding were checked to see if systematic responding was observed using two rules: (1) if the subsequent indifference point was 20% less than the preceding indifference point of the larger, later reward and (2) if the last indifference point was not less than the first indifference point by at least a magnitude equal to 10% of the larger, later reward [34].

For each participant, indifference points, or the amount of money offered now that was just as appealing to them as $100 at a future time point, were calculated for each delay. The ordinal area under the curve (AUC^ord^) was used as the measure of DD. AUC^ord^ ranges from 0 (maximum discounting of the delayed reward) to 1 (no discounting); higher values of AUC^ord^ indicate lower levels of discounting. AUC^ord^ was calculated using the ordinal values for each of the five delays. This measure was chosen over the traditional AUC measure because it does not overweight the contribution of distal delays [40].

In addition, in a sensitivity analysis, we calculated discount rates using a hyperbolic discounting model [41],

(1) $$\begin{array}{c} V=\frac{A}{1+\text{kD}}\text{,} \end{array}$$

where *V* is the discounted value, *A* is the reward amount, *D* is the delay, and *k* is a free parameter that indexes the rate of discounting. Higher values of *k* indicate more rapid devaluation of the delayed reward and greater impulsivity.

The CFCS is a 12-item scale that measures how much a person considers the long-term consequences of their current behavior [25–27]. Research has shown significant relations between DD and CFCS [42]. The consideration of future consequences is related to obesity [43, 44], such that the more you consider the future, the lower your BMI, but we could not identify any research on consideration of future consequences and food insecurity. CFCS asks how much one considers immediate (e.g., “I only act to satisfy immediate concerns, figuring the future will take care of itself”) and distal (e.g., I am willing to sacrifice my immediate happiness or well-being in order to achieve future outcomes”) outcomes [27]. In the present study, immediate items [4–6, 9–12] were reverse scored and averaged with the future items [1–3, 7, 8] for a total possible score between 1 and 5. Sample scores on the CFCS ranged from 2 to 5 (*M* = 3.8, SD = 0.61). Both the bi- and unidimensional scales are psychometrically valid and reliable [26], with a Cronbach's alpha of 0.82 [25]. Recent research has found the CFCS future factor to be redundant [26] and that the unidimensional model is more parsimonious than two discrete factors [25].

The Perceived Stress Scale (PSS) is a 10-item survey with questions that indicate how stressed an individual perceives themselves to be and how well equipped they are to deal with that stress (e.g., “In the last month, how often have you felt nervous and “stressed”?”, “In the last month, how often have you felt that you were unable to control the important things in your life?”) [45]. Answers range from “0” (never) to “4” (very often), positively stated items (e.g., 4, 5, 7, 8) are reverse-scored, and responses are summed. In the present study, PSS scores ranged from 0.00 to 38.00 (*M* = 16.23, SD = 7.41). The PSS has good test-retest reliability and criterion validity for measuring psychological stress, with Cronbach's alpha greater than 0.70 [46].

### 2.3. Analytic Plan

**Figure 1:**
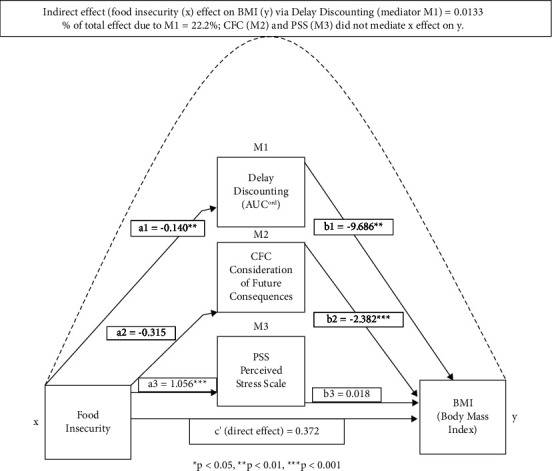
Display of the parallel influences of delay discounting, consideration of future consequences, and perceived stress on the food insecurity-BMI relation.

Preliminary analyses used zero-order correlations to explore relations among variables. To understand the income-food insecurity relation, analysis of variance (ANOVA) was used to explore the effect of income expressed as a three-category classification of low income (*N* = 53; <200% of poverty), middle income (*N* = 117; 200–399% over poverty), and higher income (*N* = 153; >400% over poverty) using 2018 standards for poverty [38] and to explore the interaction between income and DD on food insecurity. The main analytic approach was to use atemporal mediation for cross-sectional data to understand whether DD, CFCS, or perceived stress could be pathways between food insecurity and BMI, controlling for age, years of education, and percent above poverty. Atemporal mediation uses the analytic approach developed to identify mediators of change, but these methods can be used in cross-sectional research to identify relations among variables and to get a signal and potential true mediators that can be tested in future prospective research [47]. The working model that was tested is shown in Figure 1, with DD, CFCS, and stress tested for their indirect influence on food insecurity and BMI. The indirect effects and confidence intervals of DD or CFCS mediating the effect of food insecurity on BMI were first estimated in a separate analysis using Hayes process macro 4.0 [48] which constructed estimates from 10,000 bootstrap resamples. The indirect effects are presented in three ways. First, the indirect effect is significant if the bootstrapped 95% confidence interval does not contain zero. Second, the magnitude of the indirect effect reflects the estimated change in the dependent variable through the effects of the mediator variable per unit change of the predictor variable [49]. Third, the proportion of the total effect explained by the indirect effect will be quantified by the effect ratio (indirect effect divided by the total effect) [50, 51]. In addition, we assessed the distribution of food insecurity ratings and the percentage of food insecurity and high food insecurity as a function of low, middle, or high-income groups. This was accomplished using ANOVA for the food insecurity ratings and chi-square for the percentages. We also present the intercorrelations among the variables. Data were analyzed using SYSTAT 11 [52], SAS 9.4 [53], and the Hayes process macro 4.0 [48].

## 3. Results

Mothers had an average food security score of 1.02 (2.00) (mean ± SD), with 21% reporting food insecurity and 12.9% reporting high food insecurity. These numbers are greater than the 11.8% of the U.S. population reporting low food security and 4.3% of the U.S. population reporting very low food security in 2018 [54]. The percent of families receiving government assistance was 14.8%, almost all who received Supplemental Nutrition Assistance Program (SNAP) benefits (43/46), which is similar to the 11.3% of families in the United States who receive SNAP benefits [55]. The average mother had a BMI of 28.0 (7.2), with 57.8% overweight or obese (BMI ≥25) and 27.4% obese (BMI ≥30), compared to population averages in 2018 of 73.1% overweight or obese and 42.5% obese [56]. The average mother was married (77.3%), 32.7 (7.1) years of age, with 1.9 (0.9) children (see Table 1).

**Figure 2:**
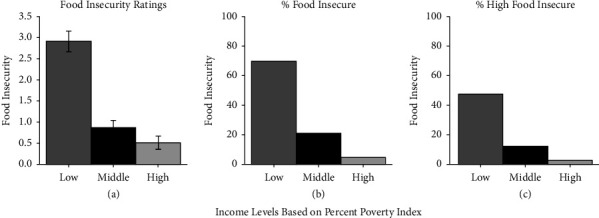
Food insecurity ratings (Mean ± SEM) (a), percent of participants with food insecurity (≥2; (b)), and percent of people with high food insecurity (≥5; (c)) in relation to income expressed as percent over poverty index adjusted for household size.

The relations among variables (Table 2) showed food insecurity was related (*p* < 0.05) to income (*r* = −0.38), education (*r* = −0.39), DD (−0.30), CFCS (−0.17), stress (0.34), and BMI (0.21). BMI was related to income (−0.15), education (−0.13), DD (−0.30), CFCS (−0.29), and stress (−0.12). DD was related to income (0.28), education (0.26), CFCS (−0.29), and stress (−0.14). The relation between income level and food insecurity ratings (Figure 2(a)), percent with food insecurity (Figure 2(b)), or percent with high food insecurity (Figure 2(c)) showed people with lower income had significantly greater food insecurity scores (*F* (2,310) = 68.98, *p* < 0.001), and a greater percentage of people with food insecurity (*X*^2^ (2) = 99.58, *p* < 0.001) or high food insecurity (*X*^2^ (4) = 101.20, *p* < 0.001). DD moderated the effect of percent over poverty on food insecurity (*F* (2,305) = 4.89, *p* = 0.008) with significantly highest food insecurity for those in low income, high DD group.

The indirect effects of food insecurity on BMI through DD, CFCS, and perceived stress are shown in Table 3 and Figure 1. The only variable that was related to both food insecurity (*p* < 0.01) and BMI (*p* < 0.001) was DD. When parallel paths were included, CFCS was related to BMI, and stress was related to food insecurity (*p*'s ≤ 0.001), but CFCS was not related to food insecurity (*p* = 0.09), and stress was not related to BMI (*p* = 0.75). The estimated indirect effect of DD on the relation between food insecurity and BMI was 0.133; 95% CI 0.206 to 0.287, the indirect effect of CFCS was 0.075, 95% CI −0.026 to 0.054, and the indirect effect of stress was 0.019, 95% CI −0.106 to 0.067. The effect ratios for the parallel effects of DD, CFCS, and stress were 22.2%, 12.5%, and 3.2% of the total observed effect of food insecurity-BMI relation on BMI, respectively (see Table 3), which accounted for 38.0 percent of the variance in the food insecurity. The pattern of results did not differ in a sensitivity analysis using log *k* as the DD measure. The indirect effect of DD on the relation between food insecurity and BMI was 0.100, 95% CI 0.014 to 0.215, the indirect effect of CFCS was 0.077, 95% CI −0.027 to 0.197, and the indirect effect of stress was 0.023, 95% CI −0.101 to 0.166. Effect ratios for the parallel effects of DD, CFCS, and stress were 16.7%, 12.8%, and 3.8% of the total observed effect of food insecurity-BMI relation on BMI, respectively.

## 4. Discussion

We found that people who discounted the future more had lower income, greater food insecurity, and higher BMI, consistent with previous research [20, 21]. The atemporal mediation model showed that DD was a potential mediator of the food insecurity and BMI relation when considered in parallel with a more general measure of prospective thinking and with perceived stress. Delay discounting has been reliably related to food insecurity [11–13, 23] and to obesity [20–22]. Having low income may shape people to allocate resources to solve immediate problems, such as paying rent, utilities, and getting food on the table. Saving and thinking about the future are luxuries that many low-income people do not experience. Paying attention to immediate needs and not focusing on an uncertain future are adaptive responses to economic scarcity [16, 17]. Disregarding immediate needs could be catastrophic for someone in a lower income bracket. In this regard, discounting the future should be considered a learned, adaptive response to their current environmental context, and not an inappropriate response for people with low income. In fact, research suggests that food insecurity is related to cognitive flexibility [57].

The narrow temporal window observed for people with food insecurity may be due in part to the unpredictable nature of access to food and meals. Basic preclinical research in different animal species has shown that animals that are provided unpredictable access to food gain more weight and find food to be more reinforcing than animals that are provided the same amount of food, but with predictable access [58, 59]. Unpredictable and irregular access to food is reliably related to food insecurity [4, 7, 59], and people with lower income may have more variability in access to food and the types of food consumed, and a strong desire to consume that food when it is available, shaping people to seek the immediate gratification associated with eating food when hungry [4, 7, 59]. Also, low income is related to a stronger reinforcing value of food [60], and unpredictable access to food makes food more reinforcing [58, 59]. Reinforcer pathology theory [61–63] integrates high food reinforcement and a short temporal window associated with immediate gratification that may help explain some of the psychological processes behind the food insecurity-obesity paradox.

The current study suggests that DD, a behavioral task designed to measure the relative strengths of immediate versus delay rewards, is more strongly related to the food-insecurity BMI relation than a more general measure of prospective thinking. We think this may be the case for two reasons. First, CFCS is a self-report measure of how people report they think about the future, as opposed to a direct behavioral measurement of this construct, and while these constructs are related, the correlation is modest. Second, the CFCS focuses on how someone might act to improve their future, but it does not compare the value of immediate versus future decisions, which is central to DD. It may be quite different to think about what you would do in the future if you have access to a very powerful, immediate reinforcer. Third, CFCS focuses on decision-making in regard to general future consequences, while the DD task involves monetary decision-making.

While stress is known to be associated with food insecurity [5, 23, 28], stress was not shown to be indirectly associated with the relation between food insecurity and BMI. This may be due to several reasons. The analytic model we used compared the parallel influence of DD, CFCS, and stress as indirect influence on food insecurity and BMI. DD may have a stronger indirect influence when stress is high. It is also possible that the influence of food insecurity on BMI is mediated by a pathway that includes stress-modifying DD which then modifies BMI, as research has shown that stress modifies decision-making [23, 32, 33]. A moderated mediator model could test if the mediating effect of discounting on BMI depends on how much stress someone is experiencing. For these reasons, we think future studies should continue to measure stress in addition to DD as part of more complex and prospective models to understand the relationship between food insecurity and BMI.

These data suggest that DD may be a novel target to reduce obesity in those with food insecurity. DD is associated with a short temporal window, and the desire for immediate gratification, rather than taking into account possibilities associated with inhibiting the immediate desires for food [18, 19]. Interventions to modify DD, such as episodic future thinking [64] or financial planning [65], aim to teach people to use prospective thinking to consider how immediate behaviors influence long-term, rather than immediate, goals. While eating a favorite food now may be very satisfying, it will not move you towards the goal of losing weight or improving your health.

As a secondary aim, we replicated the finding that income is related to food insecurity and that DD moderates the relation between low income and high food insecurity [11]. Participants with low income who did not discount the future had lower levels of food insecurity in comparison to low-income participants who did discount the future. Given that food insecurity is more common in people with low income, and low income is related to DD, future research should study a more complex model that relates income, food insecurity, DD, and BMI. Prospective research is needed to test how these variables are related. 

### 4.1. Limitations

Despite the fact that the study used a large sample and replicates previous studies showing that DD is related to both food insecurity and obesity, and DD may help explain the food insecurity-obesity paradox, there are limitations to the study. First, the study only included women, as food insecurity has a greater impact on obesity in women than men [4, 8], but research is needed on large samples of men with food insecurity. The sample was relatively high income, and predominantly white, but the percentage of families with food insecurity was representative of the percentage of families in the United States who experience food insecurity. However, given that rates of food insecurity are greater in African American than white women [66], and the reason for this relationship is poorly understood [66], it is possible that a different pattern of results would be observed if a larger percentage of African American mothers was studied.

While the atemporal mediational model suggested an influence of DD on the relation between food insecurity and BMI, the study was cross-sectional. We believe that cross-sectional studies can provide ideas for future prospective analyses of mediators, but prospective research testing whether DD mediates the relation between food insecurity and weight gain is needed. One hypothesis for a prospective study might be that low income is associated with food insecurity only when people have a narrow temporal window for decision-making and discount the future, and that discounting of the future also mediates the relation between food insecurity and weight gain. Since research has shown that low income is associated with greater food reinforcement, unpredictable access to food can increase the reinforcing value of food, including food reinforcement plus DD using reinforcer pathology theory in future research may provide greater explanatory power than DD alone. Another potential limitation is that only discounting of money was measured, and discounting of food may be a more sensitive measure for people with food insecurity, as shown by Rodriguez and colleagues [12]. However, there may be a challenge in interpreting discounting for food when studying people with obesity who know they should consume less food. Asking them whether they want a small amount of food now or a larger amount later is difficult to interpret, as a person with obesity may know they should eat less now rather than more later, and respond as if they were seeking immediate gratification, rather than just eating less. Finally, BMI was based on self-reported height and weight, and while self-report has been shown to be a valid measure of BMI in a large sample of U.S. adults [37], people do tend to overreport their height and underreport their weight, and stronger food-insecurity obesity relations may have been observed if height and weight were measured objectively. BMI also has limitations in measuring excess body fat [67], which may be particularly relevant for people with food insecurity who demonstrate substrate oxidation patterns consistent with relative carbohydrate oxidation and storage of fat [68]. 

## 5. Conclusions

Steeper discounting of the future is associated with greater food insecurity and higher BMI, and DD adds to our understanding of factors that influence the relationship between food insecurity and body weight.

## Figures and Tables

**Table 1:** Participant characteristics.

Variables	Mean ± SD or *N* (%)
Food insecurity score	1.05 ± 2.03
Low food security	65/313 (21%)
Very low food security	40/313 (12.9%)
BMI	28.00 ± 7.16
Overweight (BMI ≥25)	181/313 (57.8%)
% Obese (BMI ≥30)	89/310 (28.4%)
Income	111,057 ± 77434
Percent over poverty adjusted for household size	459.3 ± 320.4
Low income (<200% of poverty line)	53 (16.9%)
Middle income (200–399% of poverty)	107 (34.2%)
High income (greater than 400% of poverty)	153 (48.9%)
Percent on government assistance	46/313 (14.7%)
Education (years)	16.32 ± 2.48
Age	32.54 ± 7.14
Race	
American Indian	0 (0.0%)
Asian	2 (0.01%)
Black	9 (2.9%)
Native Hawaiian/PI	0 (0.0%)
White	289 (92.3%)
Multiracial	5 (0.16%)
Refused	8 (0.26)%
Marital status	
Single	18 (5.8%)
Married	242 (77.3%)
Living as married	19 (6.0%)
Divorced	32 (10.2%)
Widowed	2 (0.01%)
Number of children	1.93 ± 0.89
House size	3.87 ± 1.04
Perceived Stress Scale	16.37 ± 7.29
Delay discounting (ordinal area under the curve)	0.84 ± 0.14

**Table 2:** Relations among variables.

	Variables	1	2	3	4	5	6	7
1	Food insecurity	—						
2	Age	−0.11	—					
3	Income	**−0.38**	**0.23**	—				
4	Education	**−0.39**	**0.14**	**0.37**	—			
5	Stress	**0.34**	−0.11	**−0.19**	**−0.20**	—		
6	Delay discounting	**−0.30**	0.02	**0.28**	**0.26**	**−0.14**	—	
7	Consideration future consequences	**−0.17**	−0.04	**0.20**	**0.12**	**−0.20**	**0.30**	
8	BMI	**0.21**	0.06	**−0.15**	**−0.13**	**0.12**	**−0.30**	**−0.29**

All bold values are significant (p < 0.05).

**Table 3:** Models the indirect effect and effect ratios of delay discounting and food choice on the relation between food insecurity and BMI, controlling for education, age, stress, income, house size, and exercise.

Variables	Direct effect of food insecurity on BMI	Effect of food insecurity on BMI (total effect)	Effect of food insecurity on variable (path A)	Effect of variable on BMI (path B)	Food insecurity on BMI through variable (indirect effect)	95% CI		Effect ratio
						Lower	Upper	
Parallel indirect effects								
Delay discounting	0.372	0.600	**−0.013** ^*∗∗*^	**−9.686** ^*∗∗∗*^	0.133	0.021	0.287	0.222
CFCS	0.372	0.600	−0.032	**−2.382** ^*∗∗∗*^	0.075	−0.026	0.196	0.125
Stress	0.372	0.600	**1.056** ^*∗∗∗*^	0.018	0.019	−0.106	0.156	0.032
Total	0.372	0.600	—	—	0.228	0.010	0.486	0.380

CFCS = consideration of future consequences scale; significant paths A and B are bolded and noted by ^*∗∗*^*p* < 0.01, and ^*∗∗∗*^*p* < 0.001.

## Data Availability

Data can be made available after contacting the senior author.
